# Living with Hepatitis C Virus: A Systematic Review and Narrative Synthesis of Qualitative Literature

**DOI:** 10.1155/2017/3268650

**Published:** 2017-04-26

**Authors:** Laura E. Dowsett, Stephanie Coward, Diane L. Lorenzetti, Gail MacKean, Fiona Clement

**Affiliations:** ^1^The Department Community Health Sciences, Teaching, Research and Wellness Building, 3280 Hospital Drive NW, Calgary, AB, Canada T2N 4N1; ^2^O'Brien Institute for Public Health, Teaching, Research and Wellness Building, 3280 Hospital Drive NW, Calgary, AB, Canada T2N 4N1; ^3^Institute of Health Economics, 1200, 10405 Jasper Avenue, Edmonton, AB, Canada T5J 3N4

## Abstract

*Background and Aims*. The lived experience of HCV has not been well documented in the literature. The aim of this systematic review was to understand the experiences of living with Hepatitis C Virus (HCV).* Methods*. Five databases were searched from inception until January 19, 2015. Studies were included if they focused on adults diagnosed with HCV; reported experience living with HCV; and described original research.* Results*. 46 studies were included. Studies found that participants had reduced quality of life due to physical symptoms. Due to physical symptoms and discrimination, many participants switched to part-time work or quit their jobs. Many individuals reported negative experiences with the healthcare system; themes of feeling unsupported, not having adequate information, and not feeling involved in decisions were reported. Stigma significantly impacted those living with HCV.* Conclusions*. Published literature indicates that those with HCV often feel stigmatized and unsupported in their care, relationships, and work environments, while simultaneously coping with physical and psychological symptoms. This synthesis points to areas where greater education, compassion, and patient-centered healthcare could improve the experience of people living with HCV.

## 1. Background

Chronic Hepatitis C Virus (HCV) is a growing public health concern. HCV is transmitted through exposure to infected blood and cannot be spread through intact skin or mucous membranes [1]. Globally, there are approximately 170 million people diagnosed with chronic HCV [2, 3], with 3 to 4 million new cases per year [2]. It has been estimated that approximately 30% of individuals infected with HCV are unaware that they have the disease [4]. Approximately 350,000 deaths per year are attributed to a HCV related cause [5]. Those most at risk of contracting HCV include the following: those born between 1950 and1970, current or former intravenous drug users (IDUs), children born to HCV positive mothers, people in prison, hemodialysis patients, indigenous peoples, those from high prevalence countries, and recipients of blood, blood products, or organs prior to July 1992 [6–9].

Until recently antiviral medication, specifically pegylated interferon-*α* (PEG-INF) and ribavirin (RBV), was the current standard therapy for treating HCV [10]. These drugs are associated with severe side-effects (fatigue, headaches, fever, muscle pain, insomnia, nausea, hair loss, anorexia, depression, irritability, and joint pain [11]) and low cure rates of approximately 40–50% for those with Genotype 1 and 80% for those with Genotypes 2 and 4 [12]. The development of direct acting antivirals (DAAs) has changed the treatment landscape. In comparison to PEG-INF or RBV based treatments, DAAs have been found to have higher cure rates (90–100% viral clearance rates at 12 weeks) and virtually no side-effects [13]. Many consider these new drugs to be curative [14, 15].

Within this new paradigm, countries are reassessing their approach to HCV identification and management. Patient perspectives on living with HCV should be integral to clinical and policy decisions; patients' perspectives on access to treatment, quality of life, burden of illness, impact on productivity, and experiences with healthcare system help create informed evidence-based patient-centered healthcare. However, the lived experience of HCV from the patient perspective has not yet been comprehensively documented. Thus, the objective of this research synthesis was to document the experience of living with HCV from the perspective of those with an HCV diagnosis.

## 2. Methods

### 2.1. Data Sources

Using the ENTREQ guidelines for qualitative synthesis [16], a systematic review on patient perspectives of living with HCV was completed. MEDLINE, PubMED, EMBASE, PsychINFO, CINAHL, and SocINDEX were searched from inception until January 19, 2015. A library and information specialist developed the search strategy. Terms capturing the disease (e.g., hepatitis C, hep c, hepatitis c antigens, and hcv) were combined with terms reflecting the patient experience (e.g., attitude, health behaviour, experiences, and quality of life). These results were then focused to include only qualitative studies by using terms such as “qualitative research,” “focus groups,” and “grounded theory.” Terms were searched as both keywords and relevant database-specific subject headings. Results were filtered to exclude non-English results, nonhuman studies, and pediatric studies. No other limitations were used. The detailed search strategy is available in Appendix in Supplementary Material available online at https://doi.org/10.1155/2017/3268650.

### 2.2. Study Selection

Two trained independent reviewers screened the identified abstracts. Abstracts were assessed for relevance by using the following inclusion criteria: adult (over 18 years of age) women or men diagnosed with HCV; reporting on patient experience living with HCV; and original qualitative research. Abstracts were excluded if they did not meet the above inclusion criteria; or if they did not report findings from the patient perspective; reported only experience with treatment; presented primarily quantitative data; or if all of the participants were coinfected with another disease (i.e., Human Immunodeficiency Virus). Abstracts included by either reviewer proceeded to full-text review.

Studies that were included after the first screen were read in full text by two independent reviewers. Full texts were included if they met all of the inclusion criteria and did not meet any of the exclusion criteria presented in Table 1. Consensus was required for inclusion and disagreement was resolved through discussion or, if required, by consultation with a third reviewer. In order to ensure that all relevant articles were included, systematic reviews were hand-searched.

### 2.3. Data Extraction and Quality Assessment

Included studies were assessed for quality using the Critical Appraisal Skills Programme (CASP) checklist for qualitative literature [17]. This checklist is comprised of ten questions, each assessing areas of potential bias; for example, was a clear objective stated? Was the recruitment strategy appropriate? Was data analysis rigorous [17]? Each question is answered with “yes,” “no,” or “can't tell.” [17] Studies were not excluded based on quality.

### 2.4. Analysis

A narrative synthesis approach was used to identify and understand the key findings from the included studies. This approach is commonly used to synthesize heterogeneous literature [18], including qualitative studies. In the first stage of this narrative synthesis, independent reviewers extracted the overarching themes presented by each study in order to identify themes most frequently discussed within the included studies. Any discrepancies in the themes identified by the two reviewers were resolved through consensus. Using these overarching themes as a framework, subthemes and more detailed description were subsequently extracted from each study. Relationships between themes were explored. During data extraction, information, such as journal, study design, participant selection, participant inclusion and exclusion criteria, participant characteristics, and findings, was also extracted in duplicate from each included study.

## 3. Findings

**Figure 1:**
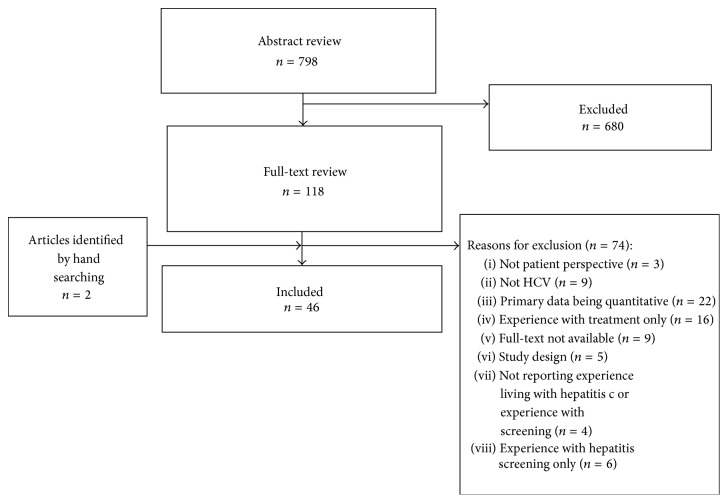
Flow chart of included and excluded studies.

Seven hundred and ninety-eight abstracts were retrieved (Figure 1). During abstract review, 118 abstracts met the inclusion criteria to continue to full-text review. Hand searching of two systematic reviews [19, 20] identified two additional studies to include in full-text review, for a total of 120 studies. Seventy-four studies were excluded for the following reasons: no patient perspective (*n* = 3), not HCV (*n* = 9), primarily quantitative data (*n* = 22), only exploring experience with treatment (*n* = 16), only focusing on experiences with HCV screening (*n* = 6), not available in full text (*n* = 9), not a relevant study design (*n* = 5), and not reporting experiences of living with HCV (*n* = 4). Forty-six studies were included in the final data set.

The included studies reported on forty-two populations (total *n* = 1619) (Table 2 and Appendix in Supplementary Material). Some of the included studies assessed the same group of people but reported on different aspects of living with HCV: Olsen et al. [21], Olsen et al. [22], and Olsen et al. [23]; Harris [24] and Harris [25]; and Tompkins et al. [60] and Wright et al. [61]. Thirty-eight studies included participants from a general population [21, 23–59], seven included participants who were IDUs [22, 60–65], and one included participants who received contaminated blood products [66]. The broad themes identified did not differ across these populations, so the findings are described together.

Seventeen studies were conducted in Australia [21–23, 25, 30, 32, 33, 38, 39, 51, 53, 56–59, 64, 65], fifteen in the UK [24, 31, 34, 35, 37, 40, 46, 47, 49, 52, 55, 60, 61, 63, 66], eight in the United States [26, 27, 36, 41, 43, 48, 54, 62], four in Canada [28, 29, 45, 50], and one each from Pakistan [42] and France [44]. The studies were published between 1999 [39] and 2014 [40, 48]. Data in these studies were analyzed using a variety of methods, including thematic and content analysis [21–23, 27, 45, 48, 56, 60, 64], inductive approach [28, 54, 57, 59], grounded theory [30, 32, 35, 46, 63], framework approach [61], phenomenological approach [31, 37, 40, 43, 51, 52, 66], interpretive approach [33, 39], and constant comparative analysis [41, 50]. Various software programs were used by these studies: NVivo [28, 29, 47, 48, 55, 62], atlas.ti [21–23, 26], and QSR*∗*NUDIST [41].

**Figure 2:**
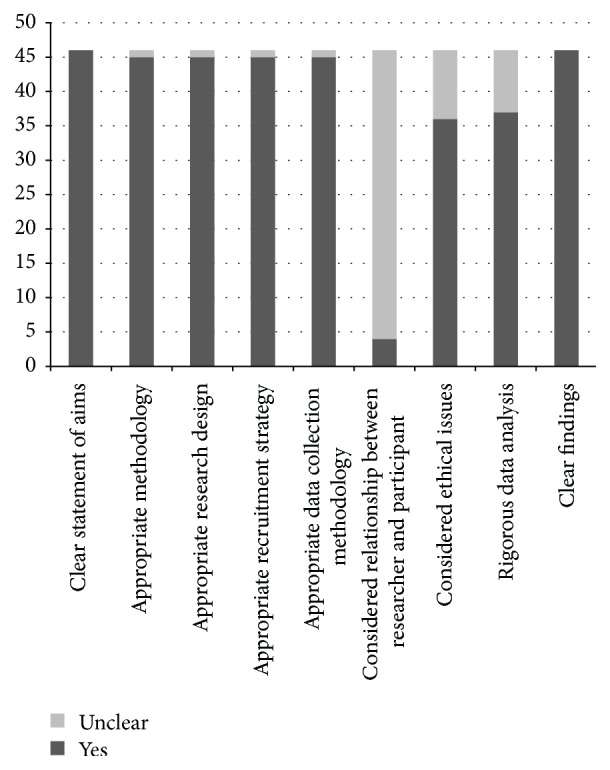
CASP Quality Assessment [22].

Broadly, the studies were of high quality; the objectives of studies were clear, as were the findings; the methods of recruitment were reasonable; and the analysis was rigorous. The areas where quality was most lacking, based on the CASP checklist, was Question 6, which asked, has the relationship between researcher and participants been adequately considered? [17] with a focus on researcher bias influencing results (Figure 2). Only four of the included studies reported any information on the relationship between researcher and participant; the remaining 42 studies were unclear. Question 7, which asked “have ethical issues been taken into consideration,” was poorly reported, with only 36 out of 46 studies reporting any information on ethical issues or ethics approval.

Initial analysis revealed five overarching themes that were frequently reported by the included studies (Box 1): disruption to daily life, communication, impact of diagnosis, preferences for care, and stigma. The key findings related to each of these five overarching themes are summarized here. The key findings related to each of these five overarching themes are summarized here.

### 3.1. Disruption to Daily Life

Broadly, studies reported that participants experienced considerable disruption to daily living, impaired quality of life, and chronic physical and psychological symptoms related to HCV. Study participants reported a variety of physical symptoms, such as fatigue [27, 30, 33–36, 40, 42, 46, 54, 62, 64, 66], weakness [27, 35], nausea [27, 30, 34, 64], pain [27, 34, 36, 42, 64, 66], swelling [27], headaches [30, 64], and sweating [30]. In addition, a number of studies reported participants experiencing psychological symptoms such as depression [27, 30, 66], anxiety or panic attacks [35], and irritability [36, 62]. Other symptoms such as poor memory [30, 35] and inability to concentrate [30, 35, 46] were also reported by participants. Fry and Bates found fatigue and other physical symptoms often made full-time work difficult to maintain; many participants switched to working part-time, quit their job, or changed sectors [33].

For participants who had undergone a liver transplant because of HCV, Dudley et al. found that they suffered from symptoms such as weight loss, lethargy, weakness, and anorexia, to the extent that, within one year of the operation, they wished that they had not received a transplant [31]. However, after one year, none of the participants regretted having the transplant [31]. Participants described that they needed “…deliberate and conscious effort to adapt to living with a transplant.” [31] Despite the long-term symptoms, participants also expressed feeling that they had a new and positive outlook on life [31].

Individuals found it difficult to abstain from alcohol or limit consumption of alcohol because their social life was tied to drinking or because they felt alienated from their social network as a nondrinker [24]. When talking about the experience of abstaining from alcohol after HCV diagnosis, patients spoke about not knowing how much alcohol they could safely consume, and receiving mixed messages from healthcare practitioners regarding alcohol consumption [24]. Some participants continued to drink heavily, often justifying their decision as prioritizing pleasure over fear of death [24].

Diagnosis of HCV was found to not significantly impact women's decisions around contraceptives [21]. Women with HCV used a variety of contraceptives or, in some cases, had reasonable justification for not using contraceptives [21]. The type of contraceptive used was dictated by women's own experiences, the experiences of other women, availability, and doctor recommendations [21].

For some, a diagnosis of HCV changes their perceptions towards injection drug use. Some people reported that, after a HCV diagnosis, a fear of transmitting HCV to others led them to develop new strategies to prevent transmission such as covering wounds, using separate injection material, and not allowing anyone to use their personal hygiene items [61]. Some participants, despite knowing that they had HCV, chose not to adopt safe injection practices to reduce transmission to others [61]. Some individuals described using more drugs after diagnosis, due to symptoms related to HCV [61].

### 3.2. Communication

Poor communication at the time of diagnosis was frequently reported. Both the level of communication and the source of the communication affected patients' perceptions of their diagnosis. One study found that the experience of diagnosis was seen in a more positive light by patients diagnosed by a family doctor or GP than those diagnosed in an organizational setting (i.e., prison and injection drug service) [56]. One participant diagnosed at a detox center said that* “he was sort of cold and…unsympathetic…it was like he does it everyday sort of thing”* and another at a prison was quoted saying that* “there's 750 prisoners. So he goes on and tells people ‘Right you've got AIDS, you've got hep C, you've got this, you've got that. Next customer'”* [56]. Participants also voiced that their experience of diagnosis was more distressing when they were not given sufficient information [25, 33, 40] or when they felt that the information was delivered insensitively (e.g., over the phone [25]).

Those with HCV expressed a desire to have involvement in decision-making regarding their care [50], to feel like healthcare practitioners were listening to their concerns [28], to feel cared for [28], and to not be spoken to insensitively [28]. Many patients believed that they were treated insensitively [40, 47, 53, 60], adequate time was not spent with them [64], confusing medical terms were used [64], and healthcare practitioners refused them treatment [47, 65].

Individuals reported that they had been given negligible information about HCV after diagnosis or were poorly informed [25, 40, 48, 59, 64], that they were told different information every visit [39], and that they were not given practical advice [40, 50]. One patient said “a lack of information from health care practitioners resulted in individuals seeking information from other sources” [48, 59]. People generally felt less stressed when more information was given [33]. One participant suggested that an education class would be valuable to those diagnosed with HCV [48]. In only one study, by Temple-Smith et al., did participants note that they felt that they had received adequate information [56].

### 3.3. Impact of Diagnosis

After receiving a positive HCV diagnosis, patients report a range of emotions, including distress (being overwhelmed and frightened and feelings of hopelessness) [25, 33, 34, 36, 39, 44, 51, 60, 62–64, 66]; shame or disgust [36, 39, 40, 44, 60]; denial or doubt [36, 44, 60]; and relief [36, 62, 66]. Studies reported that some patients were shocked or surprised by their HCV diagnosis [23, 25, 33, 34, 36, 40, 43, 44, 51, 52, 60, 63], while others were not surprised or had expected a positive HCV result [23, 33, 36, 38]. Those who were not shocked or surprised at the time of diagnosis were often current IDUs who viewed contracting HCV as inevitable [22, 23, 25, 36, 38], often thinking of HCV as a* “legacy of drug use…the junky flu, that's one of the by-products”* [40]. Some IDUs felt that they had accepted the risk of becoming HCV positive when they began using injection drugs [23, 63], as expressed by one participant* “I guess, after a while I just came to realize that if I'm going to use heroin I'll end up with hep C. It's probably inevitable…hand in hand, you know*.*”* [67] Participants that were former IDUs found that a positive HCV diagnosis was troubling because it forced them to acknowledge their previous drug use [22, 33, 67];* “I really wanted to put all that behind me…The diagnosis was ten years or whatever after I'd stopped using…I didn't really want to deal with it”* [67].

Other studies reported patients' lack of concern with their HCV diagnosis [22, 25, 33, 40, 55, 63]. This attitude appeared to stem from a variety of sources, including a lack of physical symptoms or physical impact [55] and feeling like they had bigger problems than a HCV diagnosis [22] such as a HIV diagnosis [63]. One individual was quoted as saying* “I wasn't concerned… I've got HIV anyway… just another one to add to the list”* [63].

A diagnosis of HCV impacted individuals' relationships to varying degrees. Some reported that relationships were strengthened due to the diagnosis [33, 36, 42, 52, 60, 62], while others found that HCV negatively impacted relationships [26, 30, 33, 42, 51]. Those who felt that their relationships were strengthened said that they felt more supported and closer to their family after being diagnosed with HCV [33, 36, 42, 52, 60, 62]. Those who experienced a deterioration in their relationships cited irrationality and irritability [26, 42, 51, 62], fatigue [26, 33, 51, 66], physical pain [42, 66], stress and financial burden [26], fear of transmitting HCV to family and children [30, 64], and fear of sexual transmission or pregnancy [22, 42, 47, 64], as the factors contributing to weaker relationships.

Children may be affected by their parents' diagnosis of HCV. Some individuals reported experiencing behavioural problems with their children due to the tension and deterioration of relationships between parents [51]. Some children became confused and frustrated by the symptoms their parents were exhibiting, such as lack of energy and change in their parent's behaviour [66]. One woman expressed this by saying* “my youngest…keeps asking me how old I am because I don't go here, there and everywhere with her. And she says ‘How old is that now, does that mean that you are middle aged now?'…And she would say how old is her friend's mum and she is always comparing me with her”* [66].

### 3.4. Preferences for Care

Many participants cited having a negative relationship with their healthcare providers. Examples of negative relationships included healthcare provider focusing on disease rather than the person [50, 51]; not feeling supported [42, 48, 64]; and being treated like a* “leper”* or feeling unworthy of medical care [46, 53, 56, 64, 65]. One patient was quoted as saying* “you were treated like a leper when you went into the hospital…because I had the hepatitis C, right away they think, drug addict…You know, I'd be stuck in a room and on the front of the door, it'd have ‘do not enter, infectious'”* [46].

Participants ideally wanted a health practitioner who was both well-informed and respected confidentiality. In one study, Brunings et al. reported that healthcare practitioners were poorly informed and patients felt that they were educating the doctor [28]. In another study, one patient was concerned because their healthcare practitioner had breached confidentiality [53].

Concerns about continuity of care were discussed in one study [50]. Here, participants felt that their care was fragmented, that specialists assumed that all they had to do was treat the disease and monitor disease progression, and that GPs and specialists worked in silos [50]. A patient was quoted as saying* “there isn't a network, there is just a specialist that sort of looks at one aspect of everything it seems. What I feel like sometimes is that you [health care practitioners] are building or putting together a puzzle without the picture”* [50].

Some study participants also expressed a wish for more holistic care [38, 40, 50, 51]. Participants felt that the medical model of care used by healthcare practitioners did not address all of the issues faced by those with HCV [38, 40, 50, 51].

### 3.5. Stigma

Many individuals, across these studies, described the stigma associated with HCV. The cause of stigma was consistently found to be based on either an association of HCV with injection drug use or risky behaviours [29, 30, 32, 33, 40, 41, 46, 47, 49, 51, 53, 64] and/or ignorance or misconception about transmission [26, 29, 32, 33, 41, 46–48, 53, 65]. Individuals with HCV reported that misinformation made those around them afraid of touching them [29, 41, 46, 65], and afraid of them using regular utensils and plates (e.g., those with HCV would be offered plastic utensils and paper plates) [30, 41, 46–48]. One individual said* “there's a powerlessness that goes with being represented as, you know, I'm part of the hepatitis C statistics, so when I'm reading misinformation, I feel misrepresented”* [33].

Emotional and action-based responses were reported in the included papers. Emotional responses to stigma included hurt feelings [29, 33], shame [29, 33, 49, 53], embarrassment [29, 47], low self-worth [29], fear [29, 47, 49], anger [29, 33, 49], depression [29], isolation [29], feeling dirty [37, 49, 55, 64], and feeling rejected [49]. Action-based responses to stigma included educating others [29], blaming others [29], and changing relationships [29]. Due to perceived stigma or a fear of stigmatization, those with HCV were often afraid to disclose their HCV status [37, 40, 41, 47, 49, 53, 55, 56, 64, 65], lost friends [26], changed employment to avoid stigmatization [26, 47], and isolated themselves to avoid experiencing stigma [40, 41, 46, 48, 55].

In one study, seven participants reported they had disclosed their status at work; of these, three experienced discrimination (two of whom subsequently quit due to discrimination) [33]. This finding was echoed in studies by Crockett and Gifford who found that physical symptoms and concern about disclosure were the key barriers to stable income [64] and Moore et al. who reported that the stigma of HCV deterred people from disclosing their status [47].

Study participants also reported that they experienced either overt or covert stigmatization or discrimination from healthcare practitioners [29, 38, 40, 46, 47, 53, 65]. One participant expressed that stigmatization from a healthcare professional felt particularly degrading [40]. Often, this type of stigmatization created a barrier to understanding HCV and to seeking help from healthcare professionals [33, 53, 64]. One individual was quoted as saying* “the Doctor rang me at home at 11.30pm and said ‘you've got Hep C. You're a junkie, aren't you? I know there's a lot to be ashamed of, but you can tell me'. I had never been a drug user, I had never met an intravenous drug user in my life! He said ‘you could have only got it if you were a junkie'. So I thought that was just stupid, I dismissed the diagnosis”* [33].

## 4. Discussion

A sizeable body of qualitative research was found that explored people's experiences of living with HCV, many of which included people's experiences with the healthcare system. Although the studies included a variety of population groups, common themes emerged which focused on the varying ways in which being diagnosed with and living with HCV affected people's quality of life. Both living with the symptoms of HCV and being diagnosed with HCV contributed to the impact that HCV had on people's lives. For example, the stigma associated with being diagnosed with HCV affected people's careers, relationships, and experiences with the healthcare system, as did the physical and mental health symptoms associated with HCV (e.g., fatigue, weakness, nausea, pain, depression, and anxiety).

Study participants also described having a reduced quality of life due to HCV symptoms, such as fatigue, pain, nausea, and psychological changes. Although often seen as an asymptomatic disease, these studies found that the symptoms of HCV result in considerable disruption to daily living. Three studies investigated the impact of HCV on one's career and found that, due to physical symptoms and discrimination, many participants switched to part-time work, quit their jobs, or changed employment sectors. Additionally, the published literature suggests that, for some, an HCV diagnosis strengthened their personal relationships, while, for others, it weakened relationships; symptoms of HCV (whether physical or emotional) and fear of transmission were the factors most cited for contributing to deteriorating relationships. These findings are important as they underscore that while no clinically relevant symptoms may be present, HCV can still substantially impact a patient's life.

Very few of these study participants with HCV reported positive experiences with the healthcare system. Themes of not feeling supported, stigma, not being given adequate information, and not feeling involved in decision-making were frequent in the literature. Although these represent only a small sample of individuals with HCV who interact with healthcare, these finding were generally consistent across studies, and may help practitioners better recognize the specific needs of patients with HCV. Poor experiences with diagnosis and treatment highlight the need for patient-centered care, with a focus on supporting and educating patients with HCV. For example, offering HCV education sessions to newly diagnosed patients may help patients feel more supported within the healthcare system.

Stigma significantly impacted those with HCV. Two contributing factors to HCV related stigma were consistently identified: fear of transmission and association with injection drug use or risky behaviour. Misinformation appears to be the underlying cause of this stigma. It is likely that education, within the healthcare system, for family and friends of individuals diagnosed with HCV, and public education may reduce the stigma associated with HCV. Knowledge about the causes of HCV, as well as transmission mechanisms and transmission patterns, may reduce the feelings of stigmatization felt by those diagnosed and, in turn, may have positive implications such as more interest in being tested for HCV, increased disclosure, and improved quality of life.

At the time of diagnosis, individuals can experience feelings of distress, shame, unconcern, and relief. While most experience a feeling of shock, those who are current drug users are often not surprised and see diagnosis as inevitable. Awareness of these poor experiences with diagnosis may help illuminate areas where diagnosis communication can be improved; confusion, inadequate information, diagnosis over a phone, and diagnosis in an institutional setting all lead to feelings of dissatisfaction among those with HCV. These findings should be taken into account when considering how, where, and by whom HCV screening should be done.

Two limitations of this synthesis are as follows: non-English language studies were excluded and nearly all of the included studies were conducted in English speaking countries, with a large number being completed in Australia and the UK. Two strengths are as follows: a range of countries and a variety of populations were represented in this synthesis, and there was a lot of consistency across the studies. This means that the themes identified here are likely to be transferable to similar contexts.

Additionally, by its nature, this body of literature is difficult to synthesize; there is significant heterogeneity within the studies that have been included due to the variety of at-risk population categories, the diversity of topics explored in each paper, and the vast number of experiences captured by the included papers. Although organized by theme, this systematic review does not intend to suggest that the experiences across all populations (e.g., injection drug users and general population) are the same. However, remarkable consistency of themes across all studies does suggest that different populations may have similar experiences with HCV.

## 5. Conclusions

Published literature on experiences of living with HCV indicates that those with HCV often feel stigmatized and unsupported in their care, relationships, and work environments, while simultaneously coping with a variety of physical and psychological symptoms. This synthesis points to several areas where greater education, compassion, and patient-centered healthcare could improve the experience of people living with HCV.

## Supplementary Material

The online appendix contains additional information, such as the full search strategy used for this systematic review, and a table synthesizing the characteristics of each included study.

## Figures and Tables

**Box 1:**
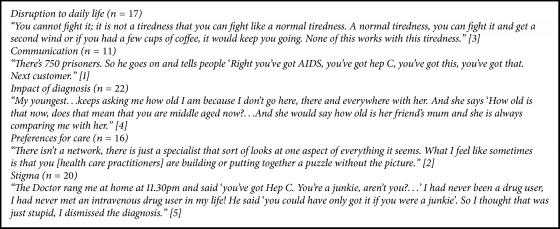
Themes explored by included studies.

**Table 1:** Inclusion and exclusion criteria for systematic review of patient experiences with HCV.

Inclusion criteria	Exclusion criteria
(i) Women or men diagnosed with HCV Virus (ii) Report on experience living with HCV from the patients perspective (iii) Original qualitative research (iv) Full-text available (v) Adult participants	(i) Individuals included in the study who do not have Hepatitis C (ii) Patient perspective (iii) Did not report experience living with HCV from the patients perspective (iv) Inclusion of participants under 18 years old (v) Physician accounts of patient experience (vi) Experience of treatment with peg-interferon medication (vii) Abstracts or posters (with no full-text available) (viii) Case studies, reviews, meta-analyses (ix) Quantitative study designs

**Table 2:** Characteristics of included studies.

	Author	Study design	Number of participants included	Themes				
				Disruption to daily life	Communication	Impact of diagnosis	Preferences for care	Stigma
General population	Blasiole et al., 2006	Semistructured interview	342			✓		✓
	Bova et al., 2008	Semistructured interviews	39	✓				
	Brunings et al., 2013	Semistructured focus group interviews	44		✓		✓	
	Butt et al., 2008	Interviews and daily think-aloud recordings	26					✓
	Conrad et al., 2006	Semistructured interviews	70	✓		✓		✓
	Dudley et al., 2007	In-depth interviews	8	✓				
	Faye and Irurita, 2003	Semistructured interviews	24					✓
	Fry and Bates, 2012	Semistructured interviews	15	✓	✓	✓		✓
	Glacken et al., 2003	In-depth interviews	28	✓				
	Glacken et al., 2001	Descriptive exploratory design	9	✓		✓		
	Groessl et al., 2008	Semistructured interviews	22	✓		✓		
	Grundy and Beeching, 2004	Semistructured interviews	8					✓
	Harris, 2010	Phenomenological research design	40	✓				
	Harris, 2005	Not reported	20			✓	✓	✓
	Harris, 2009	Semistructured interviews	40		✓	✓		
	Hepworth and Krug, 1999	Semistructured interviews	66		✓	✓		
	Hill et al., 2015	Unstructured interviews	23	✓	✓	✓	✓	✓
	Janke et al., 2008	Focus groups	40					✓
	Jiwani et al., 2013	Semistructured interviews	10	✓		✓	✓	
	Kinder, 2009	Interviews using open-ended questions	8			✓		
	Le Talec, 2013	Semistructured, open-ended interviews	31			✓		
	MacNeil, 2012	Semistructured, open-ended interviews	9			✓		
	McCreaddie et al., 2011	Semistructured interviews	16	✓			✓	✓
	Moore et al., 2009	Written questionnaire with open ended questions	39		✓	✓		✓
	North et al., 2014	Semistructured focus group interviews	48		✓		✓	✓
	Olsen et al., 2013	Semistructured, open-ended interviews	109			✓		
	Olsen et al., 2009	Semistructured, open-ended interviews	109	✓				
	Owen, 2008	In-depth, open-ended interviews	6					✓
	Paterson et al., 2006	“Think aloud” approach during a face-to-face interview	33		✓		✓	
	Sgorbini et al., 2009	Semistructured, open-ended interview	5			✓	✓	✓
	Sinclair et al., 2011	Semi-structured open-ended interview	13			✓		
	Stewart et al., 2012	Semistructured, open-ended interviews	13		✓		✓	✓
	Stoller et al., 2009	Semi-structured, open-ended interviews	42	✓				
	Sutton and Treloar, 2007	Semistructured, open-ended interviews	36			✓		✓
	Temple-Smith et al., 2004	Open-ended interview	32		✓		✓	✓
	Treloar et al., 2010	Open-ended interview	24		✓			
	Treloar and Hopwood, 2008	Open-ended interview	20		✓			
	Treloar and Hopwood, 2004	Semistructured, open-ended interviews	19		✓			

Injection drug users	Contreras and Jason, 2013	Semistructured interviews	4	✓		✓		
	Copeland, 2004	Semistructured interviews, conducted in groups			✓			
	Crockett and Gifford, 2004	Semistructured interviews	25	✓	✓	✓	✓	✓
	Habib and Adorjany, 2003	Self-reported questionnaire with open- and closed-ended questions	274		✓		✓	✓
	Olsen et al., 2012	Semistructured, open-ended interviews	83			✓		
	Tompkins et al., 2005	In-depth, open-ended interviews	17		✓	✓		
	Wright et al., 2005	In-depth interviews	17	✓				

Other	Dunne and Quayle, 2001	Semistructured focus groups	32	✓		✓		
