# Supplementary material for: Living with Hepatitis C Virus: A Systematic Review and Narrative Synthesis of Qualitative Literature
Source: Can J Gastroenterol Hepatol. 2017 Apr 26;2017:3268650. doi: 10.1155/2017/3268650 (PMC5424189; doi:10.1155/2017/3268650)
Supplement: Supplementary file 1 — The online appendix contains additional information, such as the full search strategy used for this systematic review, and a table synthesizing the characteristics of each included study. [file 3268650.f1.docx]

Online Appendix

**Search Strategy**

**MEDLINE (OVID)**

1. exp *Hepatitis C/

2. *Hepacivirus/

3. (hepatitis C or hep C or hcv or hepacivirus).ti.

4. *Hepatitis C Antigens/

5. *Hepatitis C Antibodies/

6. 1 or 2 or 3 or 4 or 5

7. limit 6 to english language

8. attitude/ or attitude to death/ or attitude to health/ or health knowledge, attitudes, practice/

9. behavior/ or health behavior/ or illness behavior/ or information seeking behavior/ or risk reduction behavior/

10. (attitude* or behavior or behaviors or behaviour or behaviours or beliefs or experiences or perception* or preference* or satisfaction or understand*).tw.

11. "Quality of Life"/

12. 8 or 9 or 10 or 11

13. 7 and 12

14. limit 13 to animals

15. limit 13 to (animals and humans)

16. 14 not 15

17. 13 not 16

18. limit 17 to (case reports or editorial or letter)

19. 17 not 18

20. exp qualitative research/

21. Focus Groups/

22. interviews as topic/ or narration/

23. interview.pt.

24. (focus group* or interview* or qualitative).tw.

25. grounded theory/

26. hermeneutics/

27. (ethnograph* or grounded theory or hermeneutic* or phenomenolog*).tw.

28. experiences.tw.

29. 20 or 21 or 22 or 23 or 24 or 28

30. 25 or 26 or 27

31. 19 and 29

32. 7 and 30

33. 31 or 32

34. limit 33 to "all adult (19 plus years)"

35. limit 33 to ("newborn infant (birth to 1 month)" or "infant (1 to 23 months)" or "preschool child (2 to 5 years)" or "child (6 to 12 years)")

36. 34 and 35

37. 35 not 36

38. 33 not 37

**PubMED**

1. Hepatitis C[MAJR]

2. *Hepacivirus[MAJR]

3. (hepatitis C or hep C or hcv or hepacivirus)[ti]

4. *Hepatitis C Antigens[MAJR]

5. *Hepatitis C Antibodies[MAJR]

6. 1 or 2 or 3 or 4 or 5

7. limit 6 to english language

8. (attitude or attitude to death or attitude to health or health knowledge, attitudes, practice)[MeSH]

9. (behavior or health behavior or illness behavior or information seeking behavior or risk reduction behavior)[MeSH]

10. (attitude* or behavior or behaviors or behaviour or behaviours or beliefs or experiences or perception* or preference* or satisfaction or understand*)[tiab]

11. "Quality of Life"[MeSH]

12. 8 or 9 or 10 or 11

13. 7 and 12

14. qualitative research[MeSH]

15. Focus Groups[MeSH]

16. (interviews as topic or narration)[MeSH]

17. interview[Publication Type]

18. (focus group* or interview* or qualitative)[tiab]

19. grounded theory[MeSH]

20. hermeneutics[MeSH]

21. (ethnograph* or grounded theory or hermeneutic* or phenomenolog*)[tiab]

22. experiences[tiab]

23. 14 or 15 or 16 or 17 or 18 or 22

24. 19 or 20 or 21

25. 13 and 23

26. 7 and 24

27. 25 or 26

**EMBASE (OVID)**

1. exp *hepatitis C/ or exp *hepatitis C antibody/ or exp *hepatitis C vaccine/ or exp *hepatitis C antigen/ or exp *Hepatitis C virus/

2. (hepatitis C or hep C or hcv or hepacivirus).ti.

3. 1 or 2

4. limit 3 to english language

5. attitude/ or attitude to death/ or attitude to health/ or attitude to illness/ or consumer attitude/ or cultural bias/ or cultural sensitivity/ or employee attitude/ or exp family attitude/ or gender bias/ or exp patient attitude/ or student attitude/

6. behavior/ or exp health behavior/ or help seeking behavior/ or illness behavior/ or motivation/

7. information seeking/

8. personal experience/

9. exp "quality of life"/

10. (attitude* or behavior or behaviors or behaviour or behaviours or beliefs or experiences or perception* or preference* or satisfaction or understand*).tw.

11. 5 or 6 or 7 or 8 or 9 or 10

12. 4 and 11

13. limit 12 to animal studies

14. limit 12 to (human and animal studies)

15. 13 not 14

16. 12 not 15

17. limit 16 to (conference abstract or editorial or letter)

18. 16 not 17

19. case report/

20. 18 not 19

21. qualitative research/ or qualitative analysis/

22. exp interview/

23. participant observation/

24. (focus group* or interview* or qualitative).tw.

25. grounded theory/

26. naturalistic inquiry/

27. phenomenology/

28. (ethnograph* or grounded theory or hermeneutic* or phenomenolog*).tw.

29. experiences.tw.

30. 21 or 22 or 23 or 24 or 29

31. 25 or 26 or 27 or 28

32. 20 and 30

33. 4 and 31

34. 32 or 33

35. limit 34 to (embryo or infant or preschool child <1 to 6 years> or school child <7 to 12 years> or adolescent <13 to 17 years>)

36. limit 34 to (adult <18 to 64 years> or aged <65+ years>)

37. 35 and 36

38. 35 not 37

39. 34 not 38

**PsycINFO (OVID)**

1. (hepatitis C or hep C or hcv or hepacivirus).ti.

2. limit 1 to english language

3. exp attitudes/

4. behavior/ or exp consumer behavior/ or exp health behavior/

5. life experiences/ or "experiences (events)"/

6. exp "quality of life"/

7. health knowledge/

8. (attitude* or behavior or behaviors or behaviour or behaviours or beliefs or experiences or perception* or preference* or satisfaction or understand*).tw.

9. 3 or 4 or 5 or 6 or 7 or 8

10. 2 and 9

11. limit 10 to animal

12. limit 10 to (animal and human)

13. 11 not 12

14. 10 not 13

15. limit 14 to (abstract collection or "column/opinion" or editorial or review-book or review-media or review-software & other)

16. 14 not 15

17. exp Case Report/

18. 16 not 17

19. limit 18 to ("0700 interview" or "0750 focus group" or 1600 qualitative study)

20. qualitative research/

21. group discussion/

22. interviews/

23. (focus group* or interview* or qualitative).tw.

24. 20 or 21 or 22 or 23

25. grounded theory/

26. phenomenology/ or hermeneutics/

27. ethnography/

28. (ethnograph* or grounded theory or hermeneutic* or phenomenolog*).tw.

29. 26 or 27 or 28

30. experiences.tw.

31. 18 and 24

32. 18 and 30

33. 2 and 29

34. 19 or 31 or 32 or 33

**CINAHL (EBSCO)**

1. ((MM "Hepatitis C") OR (MM "Hepatitis C, Chronic") ) OR TI ( (hepatitis C or hep C or hcv or hepacivirus) )  (Limit to English Language)
2. ((MH "Attitude") OR (MH "Attitude to Death+") OR (MH "Attitude to Health+") OR (MH "Attitude to Illness+") OR (MH "Attitude to Risk") OR (MH "Consumer Attitudes") OR (MH "Cultural Bias") OR (MH "Family Attitudes+") OR (MH "Gender Bias") OR (MH "Patient Attitudes") OR (MH "Student Attitudes+") OR (MH "Behavior") OR (MH "Health Behavior+") OR (MH "Harm Reduction") OR (MH "Help Seeking Behavior") OR (MH "Information Seeking Behavior") OR (MH "Risk Taking Behavior+") OR (MH "Life Experiences") OR (MH "Health Services Needs and Demand") OR (MH "Patient Satisfaction") OR (MH "Consumer Satisfaction") ) OR TI ( attitude* or behavior or behaviors or behaviour or behaviours or beliefs or experiences or perception* or preference* or satisfaction or understand* ) OR AB ( attitude* or behavior or behaviors or behaviour or behaviours or beliefs or experiences or perception* or preference* or satisfaction or understand*)
3. (MH "Quality of Life+")
4. 2 or 3
5. TI experiences OR AB experiences
6. ( (MH "Action Research") OR (MH "Ethnological Research") OR (MH "Ethnographic Research") OR (MH "Ethnonursing Research") OR (MH "Grounded Theory") OR (MH "Naturalistic Inquiry") OR (MH "Phenomenological Research") OR (MH "Qualitative Studies") ) OR TI ( (ethnograph* or grounded theory or hermeneutic* or phenomenolog*) ) OR AB ( (ethnograph* or grounded theory or hermeneutic* or phenomenolog*) )

| 1. ( (MH "Focus Groups") OR (MH "Interviews") ) OR TI ( (focus group* or interview* or qualitative) ) OR AB ( (focus group* or interview* or qualitative) ) 2. 1 and 4 and 7 3. 1 and 5 4. 1 and 6 5. 8 or 9 or 10 |  |
| --- | --- |

**SocINDEX**

1. (hepatitis C or hep C or hcv or hepacivirus)[Title]
2. (attitude* or behavior or behaviors or behaviour or behaviours or beliefs or experiences or perception* or preference* or satisfaction or understand* or quality of life)[Title/Abstract]
3. (focus group* or interview* or qualitative or ethnograph* or grounded theory or hermeneutic* or phenomenolog*)[Title/Abstract]
4. 1 and 2 and 3

Table 1: Characteristics of Included Studies on Patient Experiences Living with HCV

| **Author,**  **Year of Publication,**  **Country** | **Population** | **Study Design** | **Participant Selection** | **Participant Inclusion and Exclusion Criteria** | **Participant Characteristics** | **Findings** |
| --- | --- | --- | --- | --- | --- | --- |
| Blasiole et al.^28^  2006  United States | General Population | Semi-structured interview, analyzed using Atlas software program. | Eligible participants were consecutively recruited between October 1998 and May 2003 from a Midwestern teaching hospital during a clinic visit. | Inclusion Criteria:   - Diagnosis of HCV   Exclusion Criteria:   - Under 18 years old - prisoners - Unable to verbally communicate - Not able to provide informed consent | **342 participants included**: 37.4% female, mean age 45.2 years old (standard deviation 9.2) | This study looks at social support after diagnosis of HCV. The authors found that due to HCV, 27% of participants experienced lower levels of support, and 45% lost at least one relationship. Three key reasons for the deterioration of social support were found: a fear of transmitting HCV to others, discrimination from others, and stress due to HCV. |
| Bova et al.^29^  2008  United States | General Population | Mixed method design. Semi-structured interviews, analyzed using qualitative content analysis and qualitative descriptive methods. | Eligible participants were recruited through referral and by advertising in HIV clinics in Central and Western Massachusetts. Dates of recruitment were not reported. | Inclusion Criteria:   - Diagnosis of HCV and human immunodeficiency virus - 18 years or older - English speaking | **39 participants were included**: 46.2% female, mean age 45 years old (range 34-56, standard deviation 5) | This study found that the most frequently reported HCV symptoms were fatigue, depression, weakness, pain and swelling; and that participant had trouble differentiating between HIV and HCV symptoms. Participants controlled their symptoms through abstaining from alcohol and drugs. |
| Brunings et al.^30^  2013  Canada | General Population | Semi-structured focus group interviews were conducted, using an inductive qualitative approach. Data were analyzed using NVivo software and concept mapping. | Eligible participants were recruited from four hepatitis Clinics in British Columbia between 2001 and 2004 using referrals, posters and flyers. | Inclusion Criteria:   - Diagnosis of HCV - Use of hepatitis clinic - English speaking | **44 participants were included:** 38.6% female, mean age not reported | This study looked at care issues, and found that participants with HCV related quality of care with communication, professional competence, continuity of care, and education in order to self-manage care. This article concludes that individuals with HCV value processes more highly than outcomes and health care structures when considering quality of care. |
| Butt et al.^31^  2008  Canada | General Population | Interviews, and daily think-aloud recordings were conducted. Data were analyzed using NVivo software. | Eligible participants were recruited from two hepatology clinics and one advocacy center. Dates of recruitment were not reported. | Inclusion Criteria:   - 18 years or older - diagnosis of Chronic HCV - Can speak and understand English - Live in British Columbia   Exclusion Criteria:   - Living in an institutional care facility - Require home nursing - Cognitive or memory-deficit | **26 participants were included:** 50% female, mean age 47 years old(range: 33-76) | Findings suggest that stigma creates barriers to accessing health care, and social support. Participants reported that stigma usually stemmed from misconceptions about HCV (transmission and cause). Participants responded to stigma in a variety of ways including (but not limited to) outward anger, self-blame, embarrassment, and depression. |
| Conrad et al.^32^  2006  Australia | General Population | Semi-structured interviews were conducted. Data were analyzed using grounded-theory methods, deductive coding, and inductive coding. | Eligible participants were recruited (purposeful sample) from referrals and a community advisory group, between July 1999 and April 2000. | Inclusion Criteria:   - Residents of regional and metropolitan areas - Self-identified as having HCV - 18 years or older - HCV diagnosis at least 12 months prior to interview | **70 participants were included:** 36% female, age range from 18-60 years old. | This study identified three key themes of living with HCV: symptoms of HCV as being disruptive to daily life and work, stigma related to lack of knowledge as a widespread experience amongst participants, and fear about transmission to others. |
| Contreras et al.^62^  2013  United States | Injection Drug Users | Semi-structured interviews (18 questions) were conducted focusing on experiences of contracting and diagnosing HCV, impact of HCV, and experiences living in Oxford House. Data were analyzed using NVivo software, and coded using a hierarchical coding system. | A convenience sample of eligible women were recruited from Oxford House residents in Chicago and Illinois. Dates of recruitment are not reported | Inclusion Criteria:   - Living in an Oxford House - Diagnosis of HCV | **4 participants:** 100% female, mean age 42.7 (range and standard deviation not reported) | This study found that participants were not surprised by their diagnosis (all were infected from intravenous drug use). Participants reacted to diagnosis with depression, and in one case, relief. One participant reported symptoms of fatigue, and several mentioned long-term emotional issues. Half reported an impact on relationships. |
| Copeland et al.^63^  2004  Scotland | Injection Drug Users | Semi-structured interviews, conducted in groups, were carried out using an interpretive phenomenology method. Data were analyzed using processes consistent with the grounded theory approach. | Eligible participants were recruited (purposive sampling) by referral (General Practitioner). Dates of recruitment were not reported. | Inclusion Criteria:   - Diagnosis of HCV - Current or past injection drug use - Registered with Muirhouse Medical Practice - Member of Edinburgh Drug Addiction Society | **Sixteen participants:** 56% female, mean age for females 41 years old (range 35-40, standard deviation 5.34), mean age for males 36 years old (range 30-46, standard deviation 5.16) | Key themes related to living with HCV included: reaction to diagnosis, HCV knowledge, awareness of transmission, and meaning of HCV. Some participants were indifferent to diagnosis, some were frightened and others were less concerned with HCV than with HIV. Some expressed relief that they had a diagnosis for their health problems. Participants felt that there was a lack of information on HCV. |
| Crockett et al.^64^  2004  Australia | Injection Drug Users | Semi-structured interviews were conducted, coded using an alpha hierarchical system and analyzed using thematic and content analysis. | Eligible participants were recruited (purposive sample) near Melbourne from a Needle-Syringe Program between 1999 and 2000. | Inclusion Criteria:   - Women - Diagnosed with HCV - Current or past injection drug users | **25 participants:** 100% female, mean age 29 years old (range 18-43) | This study found that women experienced stigma that significantly impacted their lives, careers, and access to health care and social support. Most women felt they were not given enough information or support at time of diagnosis. Women felt HCV had impacted their physical and emotional health which were seen as a barrier to secure employment. |
| Dudley et al.^33^  2007  England | General Population | In-depth interviews were conducted, following a phenomenological design. | Eligible participants were recruited, using purposive sampling. Dates of recruitment are not reported, and methods of recruitment are not reported. | Inclusion Criteria:   - Had liver transplant due to HCV (at least 1 year previously) - English speaking | **8 participants were included:25% female,** mean age was 51 years old (range 44-60) | Five themes were identified: long-term physical symptoms, stigma that cause embarrassment and guilt, uncertainty around health and length of life, transplant as giving participants a new outlook, and gratitude towards the donor. |
| Dunne et al.^66^  2001  Ireland | HCV diagnosis from contaminated Anti-D Immunoglobin injection to prevent RH Haemolytic Disease | Semi-structured focus groups were conducted. Themes identified using interpretive phenomenological analysis | Participants of “Positive Action”, a hepatitis support group, volunteered to be a part of the study. Dates of recruitment and methods of recruitment are not reported. | Inclusion Criteria:   - Members of “Positive Action” - Female - HCV diagnosis, with Iatrogenic origin (from contaminated Anti-D Immunoglobin injection to prevent RH Haemolytic Disease) | **32 participants were included:** 100% women, mean age not reported (range 40-50 years old) | This study found themes of women being frustrated by not having an explanation for their symptoms prior to HCV diagnosis, relief when diagnosis was made, various emotions felt after diagnosis, concern about cognitive dysfunction and negative impact on family life. |
| Faye et al.^34^  2003  Australia | General Population | Study methodology was based on grounded theory, and constant comparative analysis was used to analyze data. | Eligible participants were recruited through advertising, and two HCV associations between 1996 and 2000. | - None reported | **24 participants were included:** sex of participants was not reported, mean age was 43 (range 21-73) | The main theme identified by this study was participants experience with “being condemned.” Depression was also a theme the emerged throughout this study. |
| Fry et al.^35^  2012  Australia | General Population | Semi-structured interviews were conducted. An interpretive approach (Berg, 2004) was used to analyze data. | Eligible participants were recruited through advertising in HCV publications, support groups and snowballing. | Inclusion Criteria:   - HCV diagnosis   Exclusion Criteria   - Major health condition, beyond HCV (haemophilia, HIV) - Diagnosed within past 12 months | **Fifteen participants were included:** 67% female, mean age 44.4 years old (range 35-51) | Key theme of empowerment through knowledge, the importance of self-care, shock and distress when diagnosed, distress from discrimination, difficulty with career due to symptoms were discussed by participants. |
| Glacken et al.^36^  2001  Ireland | General Population | Descriptive exploratory design | Eligible participants were recruited (using nominated sampling) by chairpersons of HCV support groups | - None reported | **Nine participants were included:** 78% female, mean age not reported (range 27-66) | This study reports on barriers, facilitators and indicators of health life transition with HCV. Barriers included knowledge about HCV, stigma, feeling of loss-of-self, and environment. Facilitator included social support, self-care, and positive attitude. |
| Glacken et al.^37^  2003  Ireland | General Population | In-depth interviews, using a grounded theory approach. | Eligible participants were recruited using theoretical sampling. Dates and methods of recruitment were not reported. | - None reported | **Twenty-eight participants were included:** 71% female, mean age not reported (range 36-64) | This study found themes of chronic fatigue, varying severity of fatigue from one day to the next, physical weakness, cognitive symptoms such as lack of concentration and forgetfulness, and change in mood (irritability). |
| Groessl et al.^38^  2008  United States | General Population | Semi-structured interviews with eleven questions were conducted. Data were coded by two independent researchers. | Eligible participants were recruited by referral from the San Diego HCV clinic, and through advertisement on bulletin boards between 2004 and 2006. | Inclusion Criteria:   - Diagnosis of HCV - United States Veterans - Chronic HCV | **Twenty-two participants were included:** 0% female, mean age 52.1 (standard deviation 6.1) | This study found that participants responded to diagnosis with feelings of fear, guilt, anger, depression, and shame. Participants reported receiving education on HCV, but some reported misinformation, all expressed motivation for learning more about self-management. Symptoms such as fatigue, and pain were most often reported. Impact on social support and relationships varied. |
| Grundy et al.^39^  2004  England | General Population | Semi-structured interviews were conducted. Data were analyzed using Colaizzi’s method of phenomenological analysis. | Eligible participants were recruited by chart review from two National Health Service Trusts and one drug dependency unit in England. Dates of recruitment were not reported. | - None reported | **8 participants were included:** 100% women, mean age not reported and exact range not reported **(early 20s to late 50s)** | Transmission was a significant concern for participants; five women stated that passing HCV on to their children was their biggest concern, next to dying. All felt that their diagnosis had impacted their sex lives. Seven of the eight women felt stigmatized, and because of that, they often did not disclose their disease status to others. Feelings associated with diagnosis include: fear, panic and depression. |
| Habib et al.^65^  2003  Australia | Injection Drug Users | Self-reported questionnaire with open and closed ended questions. Method of qualitative data analysis is not reported. | Eligible participants were recruited by referral and advertisement from needle and syringe programs, and one methadone clinic in Sydney, Australia from January to June 1998. | Inclusion Criteria:   - Current or past injecting drug user - Diagnosis of HCV | **274 participants were included:** 46% female, mean age 31 years old (range 17-64) | Themes of discrimination were explored in this research. More than half reported experiencing discrimination due to HCV, primarily in health care situations. Participants reported that this feeling of discrimination impacted access to health care. |
| Harris et al.^24^  2010  England | General Population | Phenomenological research design was used. Method of qualitative data collection not reported. | Eligible participants were recruited from Auckland, New Zealand and Sydney, Australia. Dates and methods of recruitment are not reported. | Inclusion Criteria:   - HCV diagnosis | **40 participants were included:** 55% female, median age 47 (range 25-63). | This study specifically explored themes of alcohol use after HCV diagnosis. Most knew that they should limit alcohol, some did not know, and only two participants were told to abstain from alcohol. Participants felt judged by others for drinking, or not drinking. |
| Harris et al.^25^  2009  Australia | General Population | Semi-structured interviews were conducted. Methods of data analysis are not reported. | Eligible participants were recruited through research notices in the New Zealand HCV Resource Center Newsletter, and through Narcotics Anonymous meetings. Participants were recruited between 2004 and 2006. | Inclusion Criteria:   - HCV diagnosis | **40 participants were included:** 55% female, median age 47 (range 25-63) | Themes that emerged in this research include: confusion over “non-A, non-B” terminology, frustration at lack of information, and HCV as less debilitating/serious compared to HIV/AIDS. Approximately half of the participants expressed concern about HCV diagnosis, while the other half were unconcerned. |
| Harris et al.^40^  2005  Australia | General Population | Methods and study design not reported | Eligible participants were recruited through research notices in the New Zealand HCV Resource Center Newsletter, and through Narcotics Anonymous meetings. | - None reported | **20 participants were included:** patient characteristics are not reported | Themes identified in this paper include: deterioration of therapeutic relationship if patient feels disrespected, lack of education on alternative medicine treatments, feeling like a number rather than a person, and poor methods of diagnosis. |
| Hepworth et al.^41^  1999  Australia | General Population | Semi-structured interviews were conducted. Data were analyzed using an interpretive interactionist approach. | Eligible participants were recruited through an HCV community-based support group. Date of recruitment not reported. | Inclusion Criteria:   - HCV diagnosis | **Six participants were included:** 50% female, mean age not reported (age range 26-48 years old) | Participants in this study reported that they felt ashamed, scared and depressed due to HCV diagnosis, they reported feelings of helplessness, panic, contamination, and fatigue. Participants reported struggling with disclosing HCV status to others, and fear of transmission. |
| Hill et al.^42^  2014  England | General Population | Unstructured interviews were conducted. A descriptive phenomenological approach was used. | Eligible participants were recruited by hepatology nurses from two large teaching hospitals in East England. | Inclusion Criteria:   - Confirmed HCV - Over 18 years old - Resident of the United Kingdom - English language speaking - Access to postal address and telephone   Exclusion Criteria:   - Current intravenous drug user - Co-infected with human immune deficiency - Active liver cancer or cirrhosis | **Twenty-three participants were included:** 52% female, mean age 28 years old. | Participants generally found HCV diagnosis ‘life changing.’ They felt that they were associated with injection drug users, regardless of their transmission route. Participants talked about both physical and psychological consequences of HCV, which in some cases led to social withdrawal and isolation. A theme of uncertainty was consistent throughout the interviews. |
| Janke et al.^43^  2008  United States | General Population | Focus groups were conducted. Data were analyzed using constant comparative analysis, using QSR*NUDIST software. | Eligible participants were recruited from outpatient liver clinics from Yale-New Haven Hospital and the Veterans Administration Connecticut Healthcare system. | Inclusion Criteria:   - Diagnosis of HCV - Undergoing treatment for HCV, finished treatment within past 6 months, or refused treatment | **40 participants were included:** sex of participants was not reported, mean age of 51.5 years old (range 40-60 years old) | Key themes identified by this study include: emotional volatility of those diagnosed with HCV, awareness of stigmatization and impact on communication and social support, and social isolation due to HCV diagnosis. |
| Jiwani et al.^44^  2013  Pakistan | General Population | Semi-structured interviews were conducted. Data were analyzed using methods developed by Morse and Field (1995). | Eligible participants were recruited using a snowball sampling method from Karachi between May and July 2010. | Inclusion Criteria:   - 18 years or older - HCV diagnosis within the past 5 years - Willingness to reflect on HCV experiences - Ability to speak English or Urdu   Exclusion Criteria:   - Comorbidities - Hospitalization at the time of interview | **10 participants were included:** 70% female, mean age not reported (range: 23-60 years old) | Themes identified by this study include: misinformation about HCV transmission (transmission through washing clothes and sharing utensils), physical suffering (pain and fatigue), emotional suffering, financial strain due to health care costs, and increased social support (from family and friends). |
| Kinder et al.^45^  2009  United States | General Population | Interviews using open-ended questions were conducted. Van Manen’s (1990) phenomenological method was used to guide the data analysis in this study. | Eligible participants were recruited (purposive sample) from support groups for HCV patients located in California. Dates of recruitment were not reported. | Inclusion Criteria:   - Male - 18-60 years old - Completed treatment for HCV with interferon alpha and ribavirin | **8 participants included:** all males, mean age not reported (range not reported). | This study found six themes regarding living with HCV: acquisition of the disease, feelings about diagnosis (confusion, surprise), treatment decision making (anxiety, fear), the “horror stories” regarding treatment, what helped (prayer, mediation, healthcare professionals, exercise), and feelings now (regret). |
| Le Talec et al.^46^  2013  France | General Population | Two semi-structured, open-ended interviews were conducted with each participant. Methods of data extraction were not reported. | Eligible participants were recruited between October 2006 and March 2008 from the HEPAIG-quali study. | Inclusion Criteria:   - HCV positive - HIV positive - Homosexual men | **31 participants included:** all male, mean age not reported (age range: 33-58 years old) | This study identified  a critical and emotional period, directly after diagnosis, during which participants were open to discuss their sexual practices and reconsider risk-reduction procedures, without being willing to give up on their satisfying sex life |
| MacNeil et al.^47^  2012  Canada | General Population | Semi-structured, open-ended interviews were conducted. Newman’s theory of health (1994) was used to as a lens for thematic analysis. | Eligible participants were recruited (convenience sample and snowballing) through a provincial HCV support group, and HCV clinic. | Inclusion Criteria:   - ≥18 years old - HCV positive diagnosis | **9 participants included:** 56% female, age range: 29-62 years old | The study identified the following themes: struggling to overcome, transcending the illness, and wanting to give  back. |
| McCreaddie et al.^48^  2011  United Kingdom | General Population | Semi-structured interviews were conducted. A constructivist grounded theory approach was used. | Eligible participants were recruited (purposive sample) from two large acute care hospitals between February and August 2008. | Inclusion Criteria:   - HCV positive diagnosis - PCR positive - Not on treatment - Varying degrees of palliation - One or more of the comorbidities: alcohol or drug abuse, mental health problems, HIV or Hepatitis B co-infection, alcoholic liver disease, diabetes   Exclusion Criteria:   - Currently on treatment - Intoxicated or incapacitated due to alcohol or drugs - Unable to consent | **16 participants include:** 15% female, mean age 49 years old (range: 34-61) | This study found that participants felt isolated due to stigma and fear of contagion. Due to stigma, some patients did not disclose their HCV status. Symptoms of fatigue or fog were often reported by participants. |
| Moore et al.^49^  2009  United Kingdom | General Population | Written questionnaire with open ended questions was used to collect data between June and August 2006. NVivo-7 was used to analyze the data. | Eligible participants were recruited (convenience sample) from liver disease support groups in a southwestern state during May 2006. | Inclusion Criteria:   - English speaking - ≥18 years old - Member of liver disease support group - HCV diagnosis | **39 participants:** 46% female, mean age 52 years old (range: 35-72) | This study identified the following themes: transmission associated stigma, disclosure associated stigma, health practice associated stigma, relationship associated stigma, and work-place associated stigma |
| North et al.^50^  2014  United States | General Population | Semi-structured focus group interviews were conducted using open-ended questions. Data were analyzed using thematic coding and content analysis. NVivo software was used for data analysis. | Eligible participants were recruited through referral by their physician, or through self-referral through advertisements in clinics. Dates of recruitment were not reported. | Inclusion Criteria:   - HCV positive diagnosis, with or without HIV co-infection - No prior or current treatment | **48 participants included:** sex of participants was not reported, mean age was 48.2 years (range 24-68 years) | This study identified the following themes: understanding of the treatment process, second hand information, social factors, need for information, treatment barriers, desire for treatment, patient’s fund of knowledge, and coping methods. |
| Olsen et al.^21^  2009  Australia | General Population | Semi-structured, open-ended interviews were conducted. Atlas.ti software was used to thematically analyze data. | Eligible participants were recruited (through purposive sampling) from various community organizations in Canberra and Melbourne Australia, between 2005 and 2006. | Inclusion Criteria:   - HCV diagnosis | **109 participants included:** 100% female, mean age 35 years old (range: 16-61) | This study identified that HCV had little impact on their contraceptive practices, but illicit drug use did. Women were more concerned in prevention of STIs than pregnancies |
| Olsen et al.^22^  2012  Australia | Injection Drug Users | Semi-structured, open-ended interviews were conducted. Data were thematically analyzed using Atlas.ti software. | Eligible participants were recruited (purposive sample) from various community organizations in Canberra and Melbourne Australia between 2005 and 2006 | Inclusion Criteria:   - Diagnosis of HCV - Women - Injection drug users | **83 participants included:** 100% female, age range:16-61 years old | This study identified the following themes: drug dependence, unstable housing, unemployment, financial strain, other health issues and relationships, with concerns for HCV status were lower than other health problems and socio-economic circumstances. |
| Olsen et al.^23^  2013  Australia | General Population | Semi-structured, open-ended interviews were conducted. Atlas.ti software was used to thematically analyze data. | Eligible participants were recruited from various community organizations in Canberra and Melbourne. | Inclusion Criteria:   - Self-report as HCV positive | **109 participants included:** 100% female, mean age not reported (range of ages not reported). | This study found that women who had not injected drugs, or those who had not injected drugs in a long time were most affected by an HCV diagnosis. Those who were current injection drug users were generally not shocked, and accepted it as inevitable, since it was normalized in their social group. |
| Owen et al.^51^  2008  United Kingdom | General Population | In-depth, open-ended interviews were conducted. Method of data analysis not reported. | Eligible participants were recruited (through snowballing) from personal contacts of the researchers. | Inclusion Criteria:   - HIV/HCV co-infection or prior treatment for HCV - Homosexual men | **6 participants included:** 0% female, mean age not reported (age range: 32-43 years old) | This study identified that the stigma associated with HCV is more pronounce than HIV among gay men. Infected men reported that disclosure of HCV status was more difficult than disclosure of HIV status, and often HCV would not be disclosed. |
| Paterson et al.^52^  2006  Canada | General Population | This study used an interpretive description design. Data was collected using a “think aloud” approach during a face-to-face interview. Data were analyzed using a constant comparative analytic approach. | Eligible participants were recruited through two gastrointestinal disease clinics associated with a tertiary care hospital, at a community hospital and through paper and online advertisements. | Inclusion Criteria:   - English speaking - ≥18 years old - No known cognitive or memory deficit - HCV positive diagnosis - Resident of British Columbia   Exclusion Criteria:   - Require home nursing or institutional care | **33 participants included:** 45% female, mean age 47 years old (range: 33-76) | This study identified the need for practitioners to treat HCV as a chronic illness, and not just in terms of the persistence of the virus. |
| Sgorbini et al.^53^  2009  Australia | General Population | Semi-structured, open-ended interview questions were conducted. This study used a Heideggerian phenomenology approach. | Eligible participants were recruited (using purposive sampling) from liver clinics and advertisements between 2004 and 2006 in Sydney Australia. | Inclusion Criteria:   - ≥18 years old - HCV positive diagnosis - On combination therapy - In a partnered relationship and living with them | **5 participants included**: mean age not reported (age range: 32-54 years old) | This study identified that HCV had an enormous impact on the lives of the patients (physiological and mental), their partners, and families |
| Sinclair et al.^54^  2011  United Kingdom | General Population | Semi-structured open-ended interview questions were conducted. Data were analyzed using an interpretative phenomenological approach (Smith, 1995). | Eligible participants were recruited, through referral by a doctor, clinic nurse, psychiatrist or psychologist, from September 2004 to October 2005 through attendance at the Royal Free Hospital. | Inclusion Criteria:   - English speaking - >18 years old - HIV/HCV co-infection - Began HCV treatment - Men who identify themselves as being homosexual   Exclusion Criteria:   - Acute psychiatric illness - Difficulty speaking English | **13 participants included:** 0% female, age range 27-46 years old | This study identified the following themes: HCV diagnosis and treatment, HCV treatment education as empowering, change in sense of self (loss of former sense of self, irritability), and sexual risk-taking. |
| Stewart et al.^55^  2012  Australia | General Population | Semi-structured, open-ended interviews were conducted. Data were analyzed following the approach developed by Braun and Clarke (2006). | Eligible participants were recruited (using purposive sampling) between May and July 2010 from a clinic at the Royal Adelaide Hospital. | Exclusion Criteria:   - Co-infection with HIV or hepatitis B virus | **13 participants included:** 38% female, mean age 50.6 years old (range not reported, sd: 13.8) | This study identified the following negative themes: impact of diagnosis, stigma, unwarranted fears regarding transmission and disease progression. The following positive theme of the information provision and access to informal and formal support. |
| Stoller et al.^56^  2009  United States | General Population | Semi-structured, open-ended interviews were conducted. Data were analyzed using an emic inductive approach. | Eligible participants were recruited from emergency departments after diagnosis with HCV and no follow-up, between 2003 and 2004. | Inclusion Criteria:   - Non-abusing drinkers | **42 participants included:** 38% female, mean age 49.5 years old (range: 36-74) | This study identified the following themes: medical self-care, behavior change, and coping. The authors correlated these themes with using them to address fighting the virus, strengthening the body and managing consequences. |
| Sutton et al.^57^  2007  United Kingdom | General Population | Semi-structured, open-ended interviews were conducted. Data were analyzed using NVIVO software. | Eligible participants were recruited through various recruitment strategies in greater Sydney including advertisement in a HCV newsletter, fliers, and through referral from a needle and syringe program. | - None listed | **36 participants included:** characteristics of participants not reported | This study identified that the social consequences of living with HCV were more significant and had greater impact than clinical markers of disease progress and should be emphasized in understandings of transformation experiences in chronic illness. |
| Temple-Smith et al.^58^  2004  Australia | General Population | Open-ended interview questions, analyzed using content and thematic analysis. | Eligible participants were recruited (through purposive sampling) through referral from needle-syringe exchanges, Liver Clinics, and HCV Council between 1998 and 1999. | - None listed | **32 participants included:** 63% female, age range from 17-56 years old | This study identified the following themes: unsatisfactory experiences at time of diagnosis, concerns about transmission, the illness experience (between men and women) and seeking health care, social support, information and care, stigma. |
| Tompkins et al.^26^  2005  United Kingdom | Injection Drug Users | In-depth, open-ended interviews were conducted. A framework approach was used to thematically analyze data. | Eligible participants were recruited (purposive sample) from a primary care centre for homeless using computerized records to identify individuals with HCV. Dates of recruitment were not reported. | Inclusion Criteria:   - Positive antibody test for HCV - Homeless | **17 participants included:** 12% female, age range: 22-49 years old | This study identified the following themes: that a positive diagnosis had lasting social, emotional, psychological, behavioural, and physical effects, and post-test discussions should be followed with additional support and counseling. |
| Treloar et al.^59^  2004  Australia | General Population | Semi-structured, open-ended interviews were conducted. Data were analyzed using an inductive thematic approach. | Eligible participants were recruited through an advertisement in The *Hep C Review* and through social networks of participants (snowballing). Dates of recruitment were not reported. | - None listed | **19 participants included:** 63% female, mean age 45 years old (range: 22-72) | This study found that non-compliance with infection control guidelines among health care workers can be identified by patients, and leads to deterring future disclosure. |
| Treloar et al.^60^  2008  Australia | General Population | Open-ended interview questions, analyzed using an inductive thematic approach. | Eligible participants were recruited from liver clinics in three tertiary hospitals in Sydney during 2004. | Inclusion Criteria:   - Minimum of 4 weeks treatment or finished within the past 6 months | **20 participants included:** 35% female, mean age 49 years old (range: 35-73) | This study identified that unrealistic optimism going into treatment can lead to unrealistic expectations and higher discontinuation. |
| Treloar et al.^61^  2010  Australia | General Population | Open-ended interview questions, analyzed using an inductive thematic approach. | Eligible participants were recruited from advertisements placed in community magazines produced by a New South Wales drug user organization and through referral from the Hepatitis Incidence and Transmission Study. Dates of recruitment were not reported. | - None listed | **24 participants included:** 38% female, mean age 35 years old (range: 21-49) | Participants in this study generally reported having a poor experience when diagnosed; feeling confused, and not being given adequate information. Participants reported that they received information and support from social groups rather than health care providers. |
| Wright et al.^27^  2005  United Kingdom | Injection Drug Users | In-depth interviews were conducted. A framework approach was used to thematically analyze data. | Eligible participants were recruited (purposive sample) from primary health care center for homeless people in the north of England. Dates of recruitment were not reported. | Inclusion Criteria:   - HCV diagnosis | **17 participants included:** sex of participants was not reported, mean age of participants was not reported (age range 22-49 years old) | This study identified that Information regarding safer and hygienic use, including accurate information regarding the most effective methods to clean used equipment, must be re-enforced by people working with homeless injecting drug users. |
